# Comparative neurologic toxicity profiles of chemotherapy versus immune checkpoint inhibitors in melanoma: a propensity score-matched analysis

**DOI:** 10.1007/s00520-026-10745-4

**Published:** 2026-05-14

**Authors:** Majd A. Abualrob, Sami Alshehab, Yousef Awad, Ranim Toma, Tarneem Azzam, Rand Abdellatif, Ali Al-Salahat, Shahd Hamid

**Affiliations:** 1 Neuroscience Institute, Hamad General Hospital, Doha, Qatar; 2 Internal Medicine, Hamad General Hospital, Doha, Qatar; 3 Arab American University, Jenin, Palestine; 4 Palestinian Ministry of Health, Ramallah, Palestine; 5 Neurology Department, Creighton University, Omaha, NE USA

**Keywords:** Melanoma, Immune checkpoint inhibitors, Chemotherapy-induced peripheral neuropathy, Immune-related adverse events, Myositis, Supportive cancer care, Real-world evidence

## Abstract

**Purpose:**

Immune checkpoint inhibitors (ICIs) have transformed melanoma treatment, yet comparative real-world data characterizing ICI-associated neurologic adverse events against the well-established neurotoxicity profile of conventional chemotherapy remain limited. We sought to contextualize the neurologic risk landscape of ICIs relative to chemotherapy as a historical comparator, to inform treatment-specific supportive care and surveillance strategies.

**Methods:**

We performed a propensity score–matched analysis using the TriNetX Global Collaborative Network, encompassing 162 healthcare organizations. Adult patients with melanoma receiving chemotherapy (dacarbazine, paclitaxel, carboplatin, or temozolomide; *n* = 9787) or ICI therapy (pembrolizumab, nivolumab, or ipilimumab; *n* = 14,627) from January 2014 through November 2025 were identified. After 1:1 nearest-neighbor matching on 52 baseline covariates, 6887 pairs were analyzed over 1–1095 days post-index therapy. Primary outcomes were peripheral neuropathy, encephalopathy, and laboratory-anchored myositis (ICD-10 myositis codes with creatine kinase ≥ 500 U/L). Fractures served as a negative control. A pre-specified conservative sensitivity analysis excluded bidirectional treatment switching, restricted the ICI cohort to anti-PD-1 predominant exposure, tightened outcome definitions, and extended follow-up to 5 years.

**Results:**

Among 13,774 matched patients (mean age 67.1 years; 48.5% male), peripheral neuropathy occurred in 7.2% of chemotherapy-treated versus 3.2% of ICI-treated patients (HR 2.47; 95% CI 2.10–2.92). Encephalopathy occurred in 1.0% versus 1.4% (HR 0.70; 95% CI 0.51–0.96). Laboratory-confirmed myositis occurred in 1.0% versus 2.1% (HR 0.47; 95% CI 0.35–0.64). No cases of Guillain–Barré syndrome or CNS vasculitis were detected. The negative control showed no significant difference (*P* = .20). In the conservative sensitivity analysis (6640 matched pairs; 5-year follow-up), the peripheral neuropathy association was substantially strengthened (HR 5.45; 95% CI 4.16–7.15) and the laboratory-anchored myositis association was essentially unchanged (HR 0.46; 95% CI 0.33–0.64), while the encephalopathy association attenuated to null (HR 1.03; 95% CI 0.79–1.34).

**Conclusions:**

Chemotherapy carries a substantially higher peripheral neuropathy risk, while ICI therapy confers elevated laboratory-confirmed myositis risk. These robust findings support treatment-specific neurologic surveillance and supportive care strategies in melanoma management, particularly neuropathy monitoring during chemotherapy and neuromuscular/CK surveillance during ICI therapy.

**Supplementary information:**

The online version contains supplementary material available at 10.1007/s00520-026-10745-4.

## Introduction

Melanoma remains a significant cause of cancer-related mortality, with approximately 100,000 new diagnoses and 8000 deaths annually in the USA [1]. The treatment landscape has been transformed over the past decade by immune checkpoint inhibitors (ICIs), which have demonstrated superior efficacy compared with traditional cytotoxic chemotherapy. Programmed death-1 (PD-1) inhibitors—pembrolizumab and nivolumab—and cytotoxic T-lymphocyte antigen-4 (CTLA-4) inhibitors, either alone or in combination, have become the standard of care for advanced melanoma [2–4].

Landmark randomized controlled trials have established the efficacy of ICIs in melanoma. The KEYNOTE-006 trial demonstrated that pembrolizumab achieved superior overall survival compared with ipilimumab, with a hazard ratio of 0.68 at final analysis [2]. The CheckMate 067 trial, with 10-year follow-up data, demonstrated that combined nivolumab plus ipilimumab achieves a median overall survival of 71.9 months compared with 19.9 months for ipilimumab monotherapy (HR, 0.53), establishing combination immunotherapy as a transformative treatment option [3, 4].

However, the improved efficacy of ICIs comes with a distinct adverse event profile characterized by immune-related adverse events (irAEs) that differ fundamentally from chemotherapy toxicities. Traditional chemotherapy agents—particularly platinum compounds and taxanes—cause dose-dependent, cumulative neurotoxic effects, with chemotherapy-induced peripheral neuropathy (CIPN) being among the most prevalent dose-limiting toxicities. A systematic review and meta-analysis of 31 trials reported CIPN prevalence of 68.1% at 1 month, declining to 30.0% at 6 months or longer post-treatment [5].

In contrast, ICIs enhance T-cell effector function by blocking inhibitory checkpoints, which can inadvertently trigger autoimmune responses affecting multiple organ systems including the nervous system and myocardium. Neurologic irAEs, though less common than dermatologic or gastrointestinal toxicities, can be severe and potentially fatal. A meta-analysis of 112 trials encompassing 19,217 patients reported fatal toxicity rates of 0.36% with PD-1 inhibitors, 1.08% with CTLA-4 inhibitors, and 1.23% with combination therapy [6]. The WHO VigiBase analysis identified ICI-associated myocarditis with mortality rates of 46% overall and 67% with combination therapy [7]. Beyond mortality, severe irAEs contribute to substantial morbidity, frequently necessitating treatment discontinuation—reported in up to 35–40% of patients receiving combination immunotherapy—and prolonged immunosuppressive management [8]. Rare cardiopulmonary toxicities, including ICI-associated pulmonary hypertension, further illustrate the breadth of potentially life-threatening complications [9]. Neurologic irAEs, while uncommon, are disproportionately associated with high-grade toxicity and treatment interruption, underscoring the need for comparative toxicity characterization [10].

Despite growing awareness of these distinct toxicity profiles, comparative data on neurologic adverse events between chemotherapy and ICIs in melanoma patients remain limited. Most evidence derives from randomized controlled trials with stringent eligibility criteria that may not reflect real-world populations, or from case series and pharmacovigilance databases that lack robust comparison groups. Real-world evidence studies using large electronic health record databases offer an opportunity to compare treatment outcomes in unselected, representative patient populations under routine clinical conditions.

Notably, conventional chemotherapy has been largely supplanted by ICIs as first-line therapy for advanced melanoma and is now primarily reserved for later lines of treatment or specific clinical scenarios [11]. Nevertheless, chemotherapy remains a well-characterized treatment modality with an established and extensively quantified neurotoxicity profile, making it a valuable historical comparator against which to benchmark and contextualize the neurologic risks specifically attributable to ICIs. Rather than guiding treatment selection between modalities that are no longer used interchangeably, such a comparison serves to quantify the distinct neurologic risk landscape of ICIs and to inform treatment-specific supportive care, surveillance, and patient counseling—aligning directly with the supportive care priorities that underpin contemporary oncology practice.

The present analysis addresses this evidence gap by leveraging the TriNetX Global Collaborative Network—a federated research network spanning 162 healthcare organizations—to compare neurologic and neuromuscular adverse events between melanoma patients treated with chemotherapy versus ICIs. We employed rigorous propensity score matching with comprehensive confounder adjustment, laboratory-anchored outcome definitions to enhance phenotype validity, negative control analyses to assess the robustness of our findings to residual confounding, and a pre-specified conservative sensitivity analysis to evaluate robustness to treatment switching, combination therapy effects, outcome phenotype specificity, laboratory threshold stringency, and external validity considerations.

## Methods

### Data source and study population

This study utilized data from the TriNetX Global Collaborative Network, which aggregates de-identified electronic health records from 162 healthcare organizations spanning the USA and international sites [12]. The network encompasses more than 90 million patient records with comprehensive clinical data including diagnoses (ICD-10 codes), procedures (CPT/HCPCS codes), laboratory results, and medications.

Adult patients (≥ 18 years) with melanoma were identified using ICD-10 diagnosis codes (C43., *D03.*) and ICD-O-3 morphology codes (8720/2, 8720/3, 8721/3, 8742/3, 8761/2, 8780/3, 872–879). The chemotherapy cohort included patients receiving dacarbazine, paclitaxel, carboplatin, or temozolomide. The ICI cohort included patients receiving pembrolizumab, nivolumab, or ipilimumab, identified by RxNorm codes or HCPCS procedure codes (J9228, J9299, C9027).

To ensure temporal alignment and consistent ICD-10 coding practices, both cohorts were restricted to patients with index events occurring on or after January 1, 2014. Patients in the chemotherapy cohort who received ICIs at any point were excluded. Patients in the ICI cohort who received chemotherapy within 3 months prior to ICI initiation were excluded to minimize combination therapy contamination. Additional exclusion criteria included multiple sclerosis, small-cell or non-small-cell lung cancer, thymoma, and metastatic disease to the brain or liver. Brain metastases were excluded because these patients frequently receive concurrent whole-brain radiation therapy, stereotactic radiosurgery, and/or prolonged corticosteroid therapy, all of which can cause neurologic complications (radiation necrosis, steroid myopathy) that would confound treatment-attributable neurotoxicity assessment [13]. Liver metastases were excluded because hepatic dysfunction can cause metabolic encephalopathy independent of treatment. Both metastatic sites are also associated with higher rates of paraneoplastic syndromes and greater disease severity heterogeneity. Although brain metastases occur in approximately 20–40% of patients with metastatic melanoma [13], this exclusion was a deliberate methodological choice to improve internal validity and strengthen causal inference; the external validity implications of this exclusion were subsequently addressed in a pre-specified sensitivity analysis (see Sensitivity Analysis below).

This study was conducted using de-identified data and was exempt from institutional review board approval under 45 CFR 46.104(d)(4). All procedures complied with HIPAA privacy regulations.

### Propensity score matching

To address confounding by indication and baseline differences between treatment groups, we performed 1:1 nearest-neighbor propensity score matching without replacement. The propensity score—the probability of receiving ICI versus chemotherapy conditional on observed baseline covariates—was estimated using logistic regression.

The propensity score model included 52 distinct covariates organized into the following domains: (1) demographics (age, sex, race, ethnicity); (2) cardiovascular comorbidities (acute myocardial infarction, heart failure, peripheral vascular disease, cerebrovascular disease); (3) pulmonary disease (COPD); (4) Metabolic disorders (diabetes mellitus, diabetes with neuropathy); (5) renal and hepatic disease; (6) baseline neurologic conditions (polyneuropathy, hereditary/idiopathic neuropathy, neuralgia); (7) functional status indicators (cachexia, malnutrition, fatigue, gait abnormalities, wheelchair dependence, supplemental oxygen dependence); (8) autoimmune conditions (thyrotoxicosis, autoimmune thyroiditis, inflammatory bowel disease, type 1 diabetes, connective tissue disorders); (9) Concurrent medications (statins, metformin); (10) laboratory values (hemoglobin, leukocyte count, neutrophil count, lactate dehydrogenase); and (11) cancer-related factors (bone metastases, radiation treatment, palliative care encounters).

Covariates were selected a priori based on clinical expertise and published literature identifying factors associated with both treatment assignment and neurologic outcomes [12]. Functional status indicators (cachexia, wheelchair dependence, supplemental oxygen) were included as proxies for Eastern Cooperative Oncology Group performance status, which is not reliably coded in administrative databases. Baseline neurologic and autoimmune conditions were included to account for pre-existing susceptibility to treatment-related neurotoxicity, and laboratory values served as surrogates for disease severity and bone marrow reserve.

Balance diagnostics were assessed using standardized mean differences (SMDs), with SMD < 0.10 indicating adequate balance. We employed an automated caliper width optimized to maximize covariate balance while retaining adequate sample size. Propensity score distributions were examined before and after matching to assess overlap and common support.

### Outcome definitions

Three primary neurologic outcomes were pre-specified: peripheral neuropathy, encephalopathy, and myositis. To enhance phenotype validity, outcome definitions were refined based on established EHR phenotyping principles, and myositis was anchored to laboratory confirmation.

Peripheral neuropathy was defined using ICD-10 codes: G62.0 (drug-induced polyneuropathy), G62.2 (polyneuropathy due to other toxic agents), G62.89 (other specified polyneuropathies), G63 (polyneuropathy in diseases classified elsewhere), G13.0 (paraneoplastic neuromyopathy and neuropathy), G56 (mononeuropathies of the upper limb), and G57.91/G57.92 (mononeuropathy of the lower limb). This broad definition was selected for the primary analysis to capture the full spectrum of neuropathic complications; however, it includes mononeuropathies and paraneoplastic codes that may not specifically reflect chemotherapy-induced peripheral neuropathy (CIPN). A restricted, treatment-attributable definition (G62.0 and G62.2 only) was applied in the sensitivity analysis.

Encephalopathy was defined using ICD-10 codes: G04.00 (acute disseminated encephalitis and encephalomyelitis, unspecified), G04.81 (other encephalitis and encephalomyelitis), G92 (toxic encephalopathy), and G13.1 (other systemic atrophy primarily affecting the central nervous system in neoplastic disease). This definition encompasses both immune-mediated encephalitis and toxic/metabolic encephalopathy; the observed encephalopathy cases therefore represent a heterogeneous phenotype that may include etiologies other than pure immune-mediated encephalitis.

Myositis was defined using a laboratory-anchored phenotype requiring both: (1) ICD-10 diagnosis codes for myositis (M60, M60.9, M33, M33.2, M33.20, M33.22, M33.13, M36.0, G72.41, G72.49) and (2) creatine kinase ≥ 500 U/L within the observation window in the primary analysis, or ≥ 1000 U/L in the sensitivity analysis. This anchored definition was designed to improve specificity by excluding nonspecific myalgia or myopathy codes without biochemical evidence of muscle inflammation [14]. The 500 U/L threshold was selected as a clinically meaningful elevation approximately 2.5–5× the typical upper limit of normal (generally 135–200 U/L in females and 170–336 U/L in males [15]); institution-specific reference ranges are not available through the TriNetX federated platform, precluding ULN-normalized analyses [16].

#### Negative control outcome

Fractures (ICD-10 codes S12, S22, S32, S42, S52, S62, S72, S82) were included as a negative control outcome [17]. Neither chemotherapy nor ICIs are expected to cause fractures; therefore, a significant difference in fracture rates between groups would suggest residual confounding or surveillance bias. An absence of difference supports the validity of propensity score matching.

The observation window extended from 1 to 1095 days (3 years) after the index event for the primary analysis and was extended to 1–1825 days (5 years) in the sensitivity analysis. Patients with the outcome of interest prior to the index event were excluded from that specific outcome analysis to isolate new-onset cases.

### Statistical analysis

Baseline characteristics were compared using chi-square tests for categorical variables and *t*-tests for continuous variables. Three complementary analytic approaches were employed for each outcome: (1) measures of association: risk differences, risk ratios (RR), and odds ratios (OR) with 95% confidence intervals; (2) time-to-event analysis: Kaplan–Meier survival curves, log-rank tests, and Cox proportional hazards regression for hazard ratios (HR); and (3) recurrence analysis: mean number of outcome episodes per affected patient. All hazard ratios are reported as chemotherapy versus ICI (i.e., HR > 1 indicates higher risk with chemotherapy; HR < 1 indicates higher risk with ICI).

To assess robustness to unmeasured confounding, we calculated *E*-values for each primary outcome [18]. The *E*-value represents the minimum strength of association that an unmeasured confounder would need to have with both the treatment and the outcome to fully explain away the observed association, conditional on measured covariates.

Two-tailed *P* values < 0.05 were considered statistically significant. All analyses were conducted using the TriNetX Analytics Platform with validated statistical modules. This study followed the STROBE (Strengthening the Reporting of Observational Studies in Epidemiology) guidelines for reporting [19].

### Pre-specified conservative sensitivity analysis

To stress-test our primary findings against potential biases—including treatment crossover, combination therapy confounding, phenotypic heterogeneity, and limited external validity—we conducted a pre-specified, conservative sensitivity analysis. This analysis modified the primary approach by (1) strictly excluding patients who crossed over between chemotherapy and immune checkpoint inhibitor (ICI) treatments at any time; (2) restricting the ICI cohort to anti-PD-1 agents (pembrolizumab or nivolumab); (3) including patients with brain or liver metastases; (4) narrowing the peripheral neuropathy definition to highly specific treatment-related ICD-10 codes (G62.0, G62.2); (5) applying a more stringent laboratory threshold for myositis (creatine kinase ≥ 1000 U/L); and (6) extending the follow-up window to 5 years (1–1825 days) to capture long-term neurotoxicity. All other analytical procedures, including 1:1 propensity score matching and outcome definitions, remained identical to the primary analysis.

## Results

### Study population

**Fig. 1:**
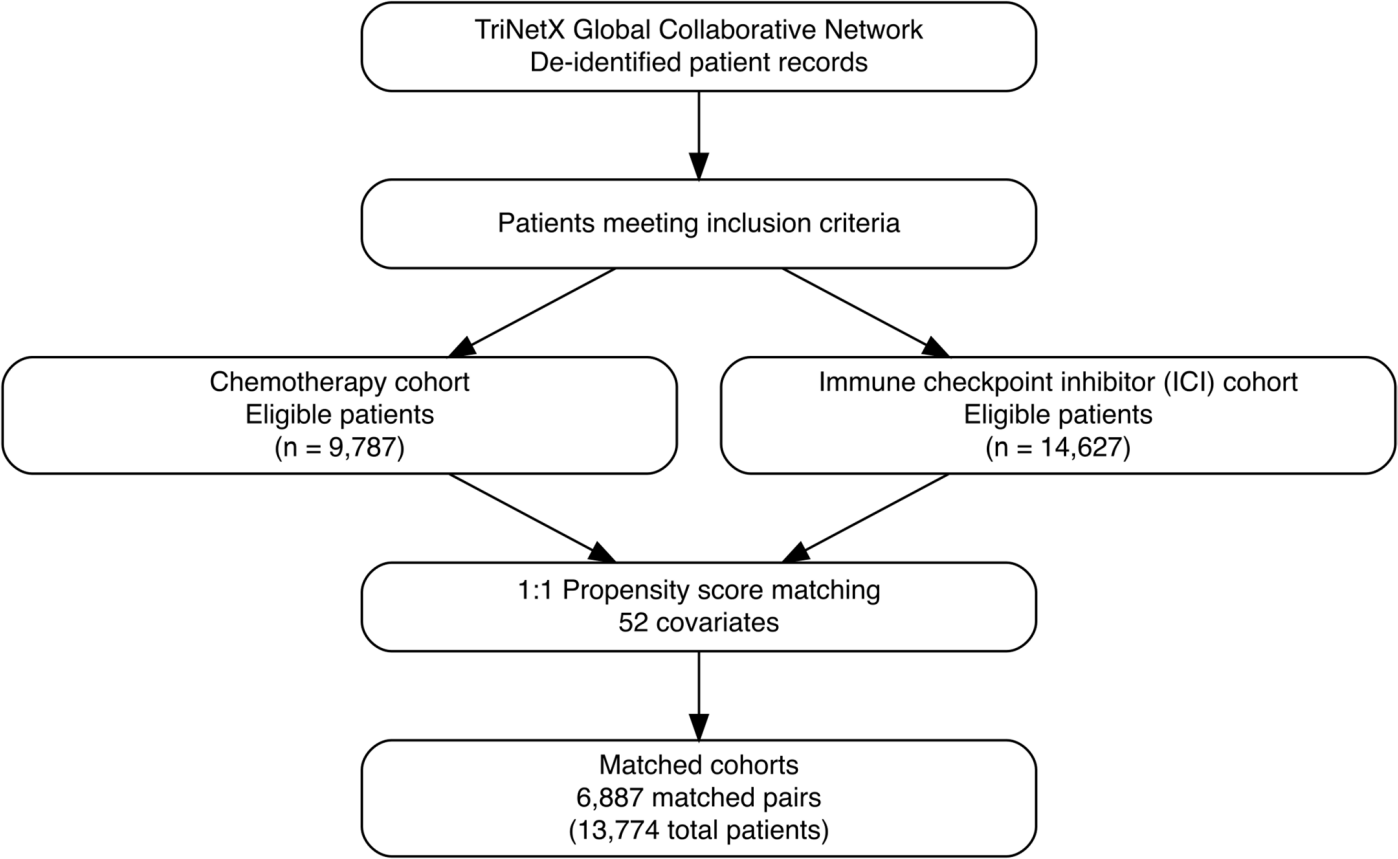
Study flow diagram. Flow diagram showing patient selection from the TriNetX Global Collaborative Network. After applying inclusion and exclusion criteria, 9787 patients in the chemotherapy cohort and 14,627 patients in the ICI cohort were eligible for analysis. Following 1:1 propensity score matching on 52 covariates, 6887 matched pairs (13,774 total patients) were included in the final analysis

Before matching, the chemotherapy cohort comprised 9787 patients, and the ICI cohort comprised 14,627 patients. After 1:1 propensity score matching, 6887 matched pairs (13,774 total patients) were available for analysis (Table 1). The matched cohorts were well-balanced on demographic characteristics: mean age was 67.1 ± 14.6 years in the chemotherapy group versus 67.0 ± 13.5 years in the ICI group (*P* = 0.945; SMD = 0.001). All covariates achieved adequate balance with SMD < 0.10 after matching. A full depiction of patient selection and matching is shown in Fig. 1.

**Table 1:** Baseline characteristics after propensity score matching (N = 6,887 per group). Demographic and clinical variables are well-balanced between the ICI and Chemo matched cohorts. Values are mean ± SD for age, and *n* (%) for categorical variables. P values and standardized mean differences (SMD) are shown for group comparisons

Characteristic	ICI (*N* = 6887)	Chemo (*N* = 6887)	*P* value	SMD
Demographics				
Age at index, years (mean ± SD)	67.0 ± 13.5	67.1 ± 14.6	0.945	0.001
Female, *n* (%)	3519 (51.1%)	3566 (51.8%)	0.423	0.014
Male, *n* (%)	3366 (48.9%)	3320 (48.2%)	0.433	0.013
White race, *n* (%)	5533 (80.3%)	5506 (79.9%)	0.564	0.010
Black or African American, *n* (%)	214 (3.1%)	196 (2.8%)	0.367	0.015
Asian, *n* (%)	82 (1.2%)	84 (1.2%)	0.876	0.003
American Indian/Alaska Native, *n* (%)	10 (0.1%)	11 (0.2%)	0.827	0.004
Native Hawaiian/Pacific Islander, *n* (%)	13 (0.2%)	13 (0.2%)	1.000	< 0.001
Other race, *n* (%)	325 (4.7%)	324 (4.7%)	0.968	0.001
Unknown race, *n* (%)	710 (10.3%)	753 (10.9%)	0.234	0.020
Not Hispanic or Latino, *n* (%)	4771 (69.3%)	4620 (67.1%)	0.006	0.047
Hispanic or Latino, *n* (%)	144 (2.1%)	149 (2.2%)	0.768	0.005
Unknown ethnicity, *n* (%)	1972 (28.6%)	2118 (30.8%)	0.006	0.046
Comorbidities and baseline diagnoses				
Brain metastasis, *n* (%)	10 (0.1%)	10 (0.1%)	1.000	< 0.001
Liver metastasis, *n* (%)	10 (0.1%)	10 (0.1%)	1.000	< 0.001
Bone metastasis, *n* (%)	135 (2.0%)	153 (2.2%)	0.284	0.018
Malignant pleural effusion, *n* (%)	42 (0.6%)	30 (0.4%)	0.156	0.024
Acute myocardial infarction, *n* (%)	347 (5.0%)	353 (5.1%)	0.816	0.004
Old myocardial infarction, *n* (%)	392 (5.7%)	392 (5.7%)	1.000	< 0.001
Heart failure, *n* (%)	700 (10.2%)	676 (9.8%)	0.495	0.012
Peripheral vascular disease, *n* (%)	385 (5.6%)	369 (5.4%)	0.549	0.010
Stroke/CVA sequelae, *n* (%)	129 (1.9%)	124 (1.8%)	0.751	0.005
Neurologic conditions at baseline				
Polyneuropathy (unspecified), *n* (%)	600 (8.7%)	549 (8.0%)	0.116	0.027
Hereditary/idiopathic neuropathy, *n* (%)	175 (2.5%)	165 (2.4%)	0.583	0.009
Neuralgia/neuritis (unspecified), *n* (%)	165 (2.4%)	157 (2.3%)	0.652	0.008
Autoimmune conditions				
Systemic connective tissue disorder, *n* (%)	172 (2.5%)	178 (2.6%)	0.745	0.006
Other systemic connective tissue involvement, *n* (%)	112 (1.6%)	108 (1.6%)	0.786	0.005
Autoimmune thyroiditis, *n* (%)	70 (1.0%)	63 (0.9%)	0.542	0.010
Crohn’s disease, *n* (%)	49 (0.7%)	53 (0.8%)	0.691	0.007
Ulcerative colitis, *n* (%)	68 (1.0%)	66 (1.0%)	0.862	0.003
Type 1 diabetes mellitus, *n* (%)	117 (1.7%)	112 (1.6%)	0.739	0.006
Diabetes mellitus (any type), *n* (%)	1355 (19.7%)	1362 (19.8%)	0.881	0.003
Chronic kidney disease, *n* (%)	926 (13.4%)	948 (13.8%)	0.585	0.009
Chronic liver disease, *n* (%)	313 (4.5%)	312 (4.5%)	0.967	0.001
Anemia (unspecified), *n* (%)	1451 (21.1%)	1433 (20.8%)	0.706	0.006

Matched cohort data from TriNetX reports. No significant differences were observed after matching (all SMD < 0.1)

### Peripheral neuropathy

Peripheral neuropathy occurred significantly more frequently in chemotherapy-treated patients. Among 6188 at-risk patients in the chemotherapy cohort and 6408 in the ICI cohort, the 3-year cumulative incidence was 7.2% (446 events) with chemotherapy versus 3.2% (205 events) with ICIs (Table 2). The risk ratio was 2.25 (95% CI, 1.92–2.65), indicating chemotherapy-treated patients had a 2.25-fold higher risk of developing peripheral neuropathy compared with ICI-treated patients.

**Table 2:** Three-year incidence of neurologic outcomes and risk estimates. Cumulative incidence at 3 years is reported for each outcome in the ICI vs Chemo cohorts, along with unadjusted risk ratios and hazard ratios (ICI relative to Chemo; RR or HR > 1 indicates higher risk with ICI) with 95% confidence intervals. Note: in the manuscript text, hazard ratios are reported as Chemo vs ICI (i.e., the reciprocal direction). *Peripheral neuropathy* and *encephalopathy* represent immune-related adverse neurologic events; *myositis* (defined by diagnostic code with supporting laboratory findings) is an immune-related myopathy outcome. *Fractures* serve as a negative control outcome. †Encephalopathy encompasses both immune-mediated encephalitis and toxic/metabolic encephalopathy (see Methods for full ICD-10 code definition)

Outcome	3-year incidence—ICI vs Chemo	Risk ratio (95% CI)	Hazard ratio (95% CI)	*P* value
Peripheral neuropathy	**3.2% vs 7.2%**	0.44 (0.38–0.52)	0.40 (0.34–0.48)	< 0.001
Encephalopathy†	**1.4% vs 1.0%**	1.47 (1.08–2.01)	1.43 (1.05–1.95)	0.02
Myositis (lab-anchored)	**2.1% vs 1.0%**	2.18 (1.63–2.93)	2.11 (1.57–2.84)	< 0.001
Fractures (negative control)	**4.6% vs 4.2%**	1.10 (0.93–1.30)	1.05 (0.85–1.30)†	0.27

No significant difference in fracture rates between cohorts (*P* = 0.27; risk ratio 1.10 ICI vs Chemo, equivalent to 0.91 Chemo vs ICI). Sources: TriNetX outcome analysis

Values in bold indicate 3-year incidence of adverse events between immune-checkpoint inhibitors and standard chemotherapy

**Fig. 2:**
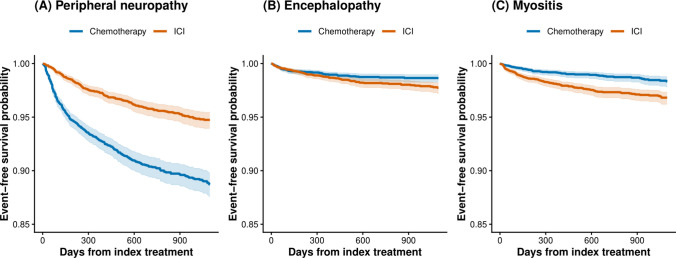
Kaplan–Meier curves for neurologic outcomes. Time-to-event analysis for **A** peripheral neuropathy, **B** encephalopathy, and **C** myositis. The *x*-axis shows days from index treatment; the *y*-axis shows event-free survival probability. Shaded areas represent 95% confidence intervals. Numbers at risk are shown below each panel. For peripheral neuropathy, chemotherapy shows significantly worse neuropathy-free survival compared with ICI. For encephalopathy and myositis, ICI shows worse event-free survival compared with chemotherapy

**Fig. 3:**
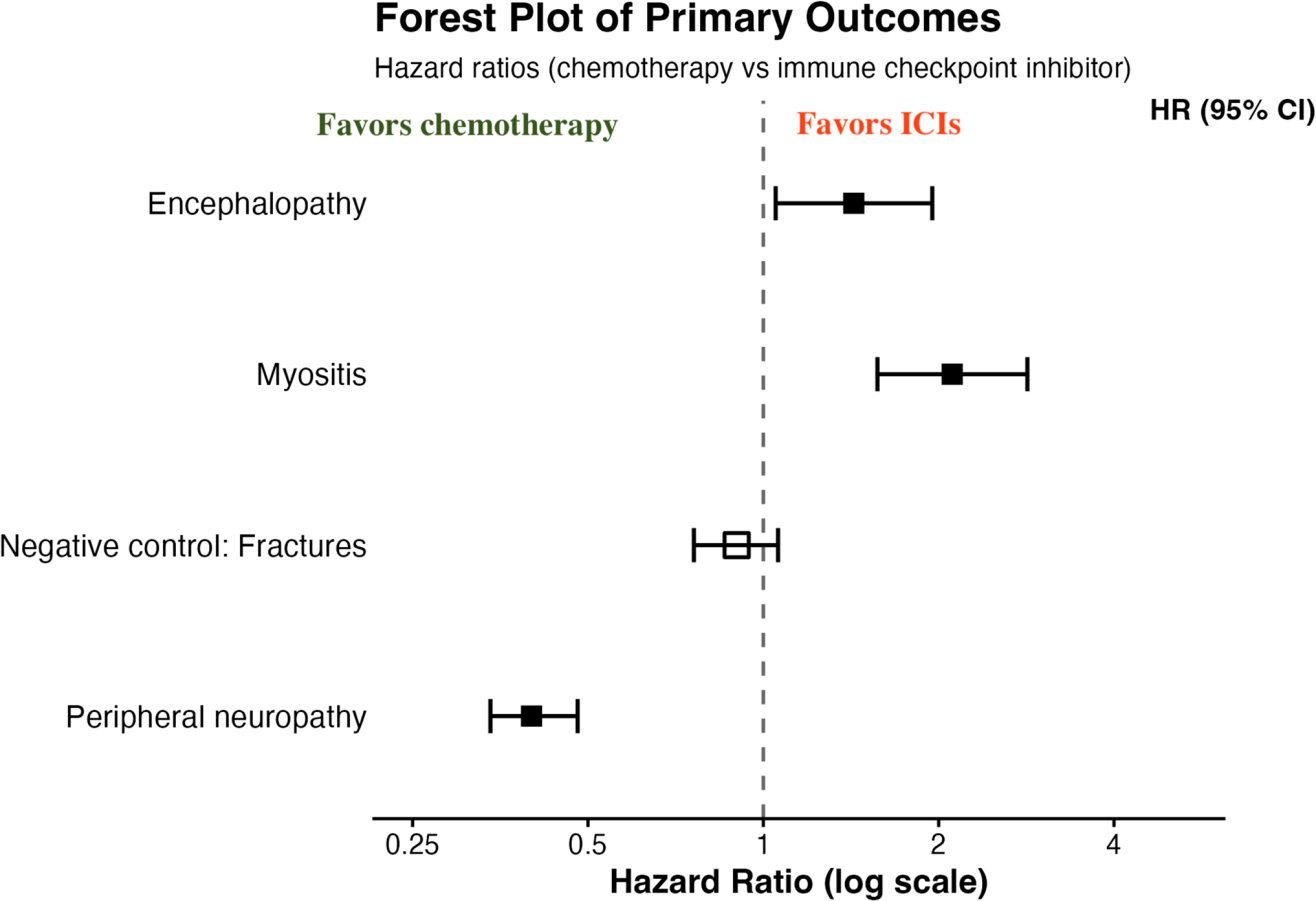
Forest plot of primary outcomes. Hazard ratios (chemotherapy vs ICI) with 95% confidence intervals for the three primary outcomes and the negative control outcome. Hazard ratios > 1 indicate higher risk with chemotherapy; hazard ratios < 1 indicate higher risk with ICI. The negative control outcome (fractures) demonstrates no significant difference between groups (HR, 0.90; 95% CI, 0.76–1.06; *P* =.20), supporting the validity of propensity score matching

Kaplan–Meier analysis demonstrated neuropathy-free survival at 3 years was 88.7% in the chemotherapy cohort versus 94.7% in the ICI cohort (log-rank *χ*^2^ = 123.1; *P* < 0.001) (Fig. 2A, Table 3). The hazard ratio for developing neuropathy with chemotherapy versus ICIs was 2.47 (95% CI, 2.10–2.92; *P* < 0.001) (Table 3, Fig. 3). The *E*-value for this association was 4.37, indicating that an unmeasured confounder would need to have a greater than fourfold association with both treatment assignment and neuropathy risk to fully explain away the observed effect (Table 4).

**Table 3:** Kaplan–Meier outcome-free survival at 3 years (ICI vs Chemo). Shown are the proportions of patients remaining free of the specified outcome at 3 years in each group (Kaplan–Meier estimates), with log-rank test statistics and hazard ratios from unadjusted survival analyses. Hazard ratios > 1 indicate faster time-to-event in the ICI group

Outcome-free Survival at 3 Years	ICI group	Chemo group	Log-Rank χ^2^ (df = 1)	Hazard Ratio (95% CI)	*P* value
Neuropathy-free survival	94.7%	88.7%	123.08	0.40 (0.34–0.48)	< 0.001
Encephalopathy-free survival	97.7%	98.7%	5.09	1.43 (1.05–1.95)	0.024
Myositis-free survival	96.8%	98.3%	25.65	2.11 (1.57–2.84)	< 0.001

Kaplan–Meier analyses of matched cohorts. Log-rank tests indicate significantly shorter event-free survival for ICI in neuropathy and myositis outcomes, and a modest difference in encephalopathy-free survival

**Table 4:** *E*-values for unmeasured confounding sensitivity analysis. *E*-values are shown for each outcome’s hazard ratio (point estimate) and for the lower bound of its 95% CI. These represent the minimum strength of association (risk ratio with both exposure and outcome) that an unmeasured confounder would need to have to explain away the observed association. Higher *E*-values indicate more robust associations less likely to be nullified by an unmeasured confounder

Outcome	Observed HR (ICI vs Chemo)	*E*-value (HR)	*E*-value (95% CI)
Peripheral neuropathy	0.40 (protective)	4.4	3.6
Encephalopathy	1.43 (harmful)	2.2	1.3
Myositis	2.11 (harmful)	3.6	2.5

For neuropathy (HR < 1), *E*-values are calculated for the equivalent HR = 1/0.40 = 2.5 (Chemo vs ICI). For encephalopathy and myositis (HR > 1), *E*-values are based on HR for ICI vs Chemo. For example, an unmeasured confounder would need a risk ratio of ≈4.4 with both treatment and neuropathy outcome to reduce the hazard ratio of 0.40 to 1.0

### Encephalopathy

Encephalopathy occurred at low rates in both groups, with a modestly higher frequency in the ICI cohort. Among 6823 at-risk patients in the chemotherapy cohort and 6833 in the ICI cohort, the 3-year incidence was 1.0% (67 events) with chemotherapy versus 1.4% (99 events) with ICIs (Table 2). The absolute risk difference was 0.4 percentage points (95% CI, 0.1–0.8; *P* = 0.013). The risk ratio was 0.68 (95% CI, 0.50–0.92), indicating a modestly higher risk with ICI therapy.

Kaplan–Meier analysis showed encephalopathy-free survival at 3 years of 98.7% in the chemotherapy cohort versus 97.7% in the ICI cohort (log-rank *χ*^2^ = 5.09; *P* = 0.024) (Fig. 2B, Table 3). The hazard ratio for chemotherapy versus ICI was 0.70 (95% CI, 0.51–0.96; *P* = 0.014) (Table 3, Fig. 3). The *E*-value for this association was 2.06. The modest effect size and lower *E*-value compared with other outcomes suggest that the difference in encephalopathy rates, while statistically significant, may be partially influenced by surveillance bias or residual confounding (Table 4)—a concern further supported by the sensitivity analysis (see below).

### Myositis (laboratory-anchored)

Using the laboratory-anchored phenotype (ICD-10 myositis codes plus creatine kinase ≥ 500 U/L), myositis occurred significantly more frequently with ICI therapy. Among 6666 at-risk patients in the chemotherapy cohort and 6624 in the ICI cohort, the 3-year incidence was 1.0% (64 events) with chemotherapy versus 2.1% (139 events) with ICIs (Table 2). The risk ratio was 0.46 (95% CI, 0.34–0.61), indicating a 2.2-fold higher risk with ICI therapy.

Kaplan–Meier analysis showed myositis-free survival at 3 years of 98.3% in the chemotherapy cohort versus 96.8% in the ICI cohort (log-rank *χ*^2^ = 25.7; *P* < 0.001) (Fig. 2C, Table 3). The hazard ratio for chemotherapy versus ICI was 0.47 (95% CI, 0.35–0.64; *P* < 0.001) (Table 3, Fig. 3). The *E*-value was 3.64 (Table 4).

### Rare neuroimmune complications

We examined Guillain–Barré syndrome, demyelinating diseases, and CNS vasculitis as exploratory secondary outcomes. No cases of any of these conditions were detected in either the chemotherapy or ICI cohort during the 3-year observation window, precluding statistical comparison. This finding may reflect both the true rarity of these conditions in the melanoma population and the limited sensitivity of ICD-10 coding for rare neurologic diagnoses, which may result in systematic underascertainment in electronic health record–based studies [20].

### Cox proportional hazards analysis: ICI class comparison

To determine whether myositis risk differed among specific ICI agents, we performed Cox proportional hazards regression incorporating pembrolizumab, nivolumab, and ipilimumab as covariates while adjusting for demographics, comorbidities, and baseline autoimmune conditions (Table 5). Treatment assignment (chemotherapy vs ICI cohort) remained the strongest predictor of myositis risk (HR, 0.56; 95% CI, 0.42–0.74; *P* < 0.001), confirming the primary analysis.

**Table 5:** Multivariable Cox Proportional Hazards Model for Myositis (Full Cohort). Cox regression results for time to myositis are shown for the full ICI (N = 14,627) and Chemo (N = 9,787) cohorts. The model includes treatment cohort and baseline covariates. Hazard ratios > 1 indicate increased hazard of developing myositis

Covariate	Hazard ratio (95% CI)	*P* value
ICI therapy (vs Chemo)	1.80 (1.34–2.41)	< 0.001
Male sex (vs female)	1.01 (0.77–1.32)	0.94
Age at index, per year	1.00 (0.99–1.01)	0.55
White race (vs other/unknown)	1.55 (0.88–2.74)	0.13
Black or African American (vs other)	0.79 (0.22–2.79)	0.71
Hispanic or Latino (vs non-Hispanic)	1.19 (0.51–2.78)	0.68
Other race (vs reference)**	1.58 (0.64–3.87)	0.99
Asian (vs reference)**	1.19 (0.27–5.29)	0.82
Autoimmune thyroiditis	1.44 (0.53–3.89)	0.48
Systemic connective tissue disorder (e.g., lupus)	4.60 (2.95–7.17)	< 0.001
Diabetes mellitus (any)	1.24 (0.89–1.73)	0.21
Hypertension	1.16 (0.86–1.58)	0.33
Heart failure	0.90 (0.54–1.48)	0.66

Hazard ratio < 1 for ICI vs Chemo indicates lower myositis hazard with Chemo (HR 0.556 for Chemo vs ICI, i.e., ICI associated with ~ 80% higher hazard). No significant associations with myositis were found for sex, age, race, or most comorbidities. However, a history of systemic connective tissue disorders (e.g., rheumatoid arthritis or systemic lupus) was associated with a markedly elevated myositis risk (HR ~ 4.6, *P* < 0.001). Reference categories: female sex; non-white/unknown race; non-Hispanic ethnicity; absence of specified condition (covariates for individual ICI agents were included in the model but are not shown; none were significant)

Individual ICI agents showed no significant differential association with myositis: pembrolizumab (HR, 0.97; 95% CI, 0.40–2.36; *P* = 0.94) and ipilimumab (HR, 1.55; 95% CI, 0.22–11.09; *P* = 0.66). The model for nivolumab did not converge, likely due to limited events within this subgroup; therefore, agent-specific risk for nivolumab could not be estimated with precision. These results suggest that the elevated myositis risk observed with ICI therapy represents a class effect rather than being attributable to any specific checkpoint inhibitor, though this interpretation should be tempered by the limited power for individual agent comparisons.

### Negative control outcome

The negative control analysis using fractures demonstrated no significant difference between treatment groups. Among 6099 at-risk patients in the chemotherapy cohort and 6271 in the ICI cohort, the 3-year fracture incidence was 4.2% (255 events) versus 4.7% (292 events). The risk ratio was 0.90 (95% CI, 0.76–1.06; *P* = 0.20) (Table 2, Fig. 3). This null finding supports the validity of our propensity score matching.

### Conservative sensitivity analysis

Under the pre-specified, maximally conservative sensitivity analysis (Supplementary Table S1)—which excluded bidirectional treatment switching, restricted the ICI cohort to anti-PD-1 therapies, included brain/liver metastases, restricted peripheral neuropathy to specific codes (G62.0, G62.2), raised the myositis threshold (CK ≥ 1000 U/L), and extended follow-up to 5 years—6640 matched pairs were available. Adequate balance was confirmed, with all 52 baseline covariates achieving SMDs < 0.10 and excellent propensity score overlap.

Under these stringent criteria, the peripheral neuropathy signal substantially strengthened. Chemotherapy demonstrated a 5.45-fold higher hazard versus ICI (304 vs 63 events; 5-year cumulative incidence 4.7% vs 1.0%; HR 5.45, 95% CI 4.16–7.15; log-rank *χ*^2^ = 189.12; *P* < 0.001), more than doubling the primary analysis magnitude (HR 2.47). Five-year neuropathy-free survival was 92.2% (chemotherapy) versus 98.2% (ICI).

Conversely, the myositis finding remained practically unchanged, confirming its robustness. ICI therapy maintained a 2.18-fold higher hazard (51 vs 119 events; 5-year cumulative incidence 0.8% vs 1.8%; HR 0.458, 95% CI 0.330–0.635; log-rank *χ*^2^ = 22.92; *P* < 0.001), closely mirroring the primary HR of 0.47. Five-year myositis-free survival was 98.5% (chemotherapy) versus 97.0% (ICI).

The fracture negative control remained null with even tighter balance (RR 0.98, 95% CI 0.83–1.16; *P* = 0.812).

Notably, the modest encephalopathy signal from the primary analysis (HR 0.70, 95% CI 0.51–0.96) attenuated completely to the null (104 vs 111 events; 5-year cumulative incidence 1.6% vs 1.7%; HR 1.03, 95% CI 0.79–1.34; log-rank *χ*^2^ = 0.04; *P* = 0.844). This supports our initial cautionary interpretation: the broad encephalopathy phenotype (pooling immune-mediated codes with toxic/metabolic code G92) is susceptible to residual confounding, rendering this conservative null finding the more reliable estimate.

## Discussion

In this propensity score–matched analysis of 13,774 melanoma patients from 162 healthcare organizations, we observed distinct neurologic adverse event profiles between patients treated with cytotoxic chemotherapy and those receiving immune checkpoint inhibitors. Chemotherapy was associated with a substantially higher incidence of peripheral neuropathy (HR, 2.47), whereas ICIs carried a higher risk of laboratory-confirmed myositis (HR, 0.47). A modest signal for encephalopathy with ICI therapy (HR, 0.70) was attenuated to the null in the conservative sensitivity analysis, suggesting that this finding should be interpreted with caution. These findings highlight the divergent mechanisms of neurologic toxicity between the two treatment modalities and underscore the importance of tailored surveillance and supportive care strategies.

The nearly 2.5-fold higher risk of peripheral neuropathy with chemotherapy is consistent with the well-established neurotoxic effects of traditional agents used in melanoma, including platinum compounds and taxanes [21]. These agents cause dose-dependent axonal damage through mechanisms including mitochondrial dysfunction and microtubule disruption. Our observed 3-year incidence of 7.2% is lower than some historical estimates, which report CIPN in up to 68% of patients at 1 month post-treatment [5]. This difference likely reflects the heterogeneity of chemotherapy regimens, variable follow-up durations, and—importantly—the reliance on diagnostic codes rather than systematic neurologic assessment in our study. Indeed, EHR-based CIPN ascertainment using ICD-10 codes captures approximately 5–26% of true CIPN cases, representing a 4–20-fold underestimate compared with prospective clinical assessment [20, 22]. The strong *E*-value of 4.37 suggests this association is robust to unmeasured confounding, and the substantially strengthened association in the sensitivity analysis (HR 5.45) using a restricted drug-specific phenotype confirms the robustness of this finding.

The elevated risk of immune-mediated neurologic complications with ICIs aligns with the mechanism of these agents, which enhance T-cell effector function and can inadvertently trigger autoimmune responses [23]. ICI-associated myositis, in particular, has emerged as a recognized toxicity that can overlap with myocarditis and carry significant morbidity [24]. Our laboratory-anchored phenotype requiring both diagnostic codes and CK ≥ 500 U/L was designed to improve specificity; the observed 2.1% 3-year incidence exceeds estimates from clinical trials (typically < 1%), possibly reflecting broader capture of subclinical or mild cases, longer follow-up, or real-world patient heterogeneity. The conservative sensitivity analysis using CK ≥ 1000 U/L and anti-PD-1 predominant exposure preserved the 2.18-fold myositis risk, providing compelling evidence that this signal is not an artifact of laboratory threshold selection or pooling of combination immunotherapy.

The absence of significant differences in myositis risk among individual ICI agents suggests myositis represents a class effect. However, this interpretation must be tempered by several considerations: the model for nivolumab did not converge due to limited events, and we could not differentiate monotherapy from combination ICI therapy in the primary analysis. In real-world practice, approximately 20–30% of first-line ICI-treated melanoma patients receive combination nivolumab plus ipilimumab [25, 26], and pooling these regimens with monotherapy in our primary analysis may have inflated toxicity estimates in the ICI arm. The conservative sensitivity analysis—which excluded ipilimumab-only monotherapy patients by restricting ICI inclusion to anti-PD-1 agents—yielded essentially identical myositis estimates (HR 0.458 vs primary 0.47), indicating that the elevated myositis risk is not driven solely by ipilimumab-containing regimens.

The higher encephalopathy rate with ICIs in the primary analysis (1.4% vs 1.0%; HR, 0.70) should be interpreted with caution, and the null finding in the conservative sensitivity analysis (HR 1.03, *P* = 0.844) strongly supports this caveat. Our encephalopathy definition combined immune-mediated encephalitis codes with toxic encephalopathy (G92), creating a heterogeneous phenotype. The modest primary effect size, lower *E*-value (2.06), and attenuation under conservative specification together suggest that this finding may have been partially influenced by residual confounding from disease severity, surveillance bias, or hepatic/metabolic encephalopathy in patients with visceral metastases (who were included in the sensitivity analysis but excluded in the primary). More refined phenotyping—for example, requiring steroid treatment or cerebrospinal fluid analysis within a defined window—could better isolate true immune-mediated encephalitis in future studies.

The null finding for our negative control outcome (fractures) in both the primary and sensitivity analyses supports the validity of our propensity score matching approach. Neither chemotherapy nor ICIs are expected to directly cause fractures, and the absence of a significant difference suggests that major confounding by health status or frailty was adequately addressed across both analytical specifications.

### Incremental contribution

Several features of this analysis extend beyond confirming known toxicity patterns. First, this is, to our knowledge, the largest propensity score–matched study directly comparing neurologic adverse events between chemotherapy and ICIs in melanoma, providing population-level risk estimates (HR 2.47 for neuropathy and HR 2.11 for myositis in the primary analysis; HR 5.45 for neuropathy and HR 2.18 for myositis in the conservative sensitivity analysis) not previously available from real-world data. Existing large-scale real-world evidence studies have compared ICI monotherapy versus combination therapy for neurologic adverse events [27] or have characterized irAE profiles using pharmacovigilance databases without comparator arms [28, 29]; however, no prior matched-cohort EHR study has directly benchmarked ICI-associated neurologic toxicity against chemotherapy in this population.

Second, the substantial gap between our EHR-based 3-year neuropathy incidence (7.2%) and trial-based CIPN estimates (30–68%) [5, 22] quantifies the magnitude of neuropathy underascertainment in administrative databases, with important implications for interpreting all EHR-based neurotoxicity studies and for designing supportive care surveillance protocols.

Third, the laboratory-anchored myositis phenotype requiring both ICD-10 codes and CK ≥ 500 U/L (or ≥ 1000 U/L in sensitivity analysis) provides a replicable, higher-specificity approach for myositis ascertainment in federated EHR databases, offering a methodological model for future pharmacoepidemiologic studies.

Fourth, the pre-specified conservative sensitivity analysis—which simultaneously addresses treatment switching, combination therapy confounding, outcome phenotype specificity, laboratory threshold stringency, and external validity concerns through bidirectional contamination exclusion, anti-PD-1 predominant restriction, inclusion of visceral metastases, restricted outcome definitions, stringent lab thresholds, and extended 5-year follow-up—represents a level of analytical robustness rarely applied to TriNetX-based studies and provides strong triangulation of the principal findings.

Finally, the negative control analysis using fractures strengthens causal inference beyond standard propensity score–matched designs, a methodological safeguard not employed in prior comparable analyses.

### Clinical and supportive care implications

As chemotherapy serves as a well-characterized historical benchmark, the comparative risk estimates provided here can inform treatment-specific neurologic surveillance and supportive care protocols in melanoma management. Patients receiving conventional chemotherapy warrant close monitoring for symptoms of peripheral neuropathy, with baseline and serial neurologic assessments including validated patient-reported outcome measures such as EORTC QLQ-CIPN20. Dose modifications, treatment delays, or therapeutic substitutions should be considered for moderate-to-severe neuropathy to preserve quality of life and functional status. In contrast, patients receiving ICIs warrant vigilance for immune-mediated neuromuscular complications, particularly myositis. Routine baseline and periodic CK measurement, coupled with clinical assessment for new-onset muscle weakness, dyspnea, dysphagia, ptosis, or altered mental status, should be incorporated into ICI monitoring protocols. New neurologic symptoms in ICI-treated patients should prompt neurology consultation and consideration of glucocorticoids or other immunosuppressive therapy guided by current irAE management guidelines [8].

### Limitations

This study has several important limitations that should be considered when interpreting the findings.

First, as an observational study using electronic health record data, there is potential for residual confounding despite propensity score matching with 52 covariates. The *E*-values provide a quantitative assessment of robustness to unmeasured confounding, but cannot exclude bias from unmeasured factors such as performance status, tumor burden, or physician treatment preferences.

Second, outcome ascertainment relied on ICD-10 diagnostic codes, which may result in misclassification. The primary peripheral neuropathy definition included mononeuropathies and paraneoplastic codes that may not specifically reflect chemotherapy-induced peripheral neuropathy; the sensitivity analysis addressed this limitation using a restricted drug-specific phenotype, which strengthened the finding. The encephalopathy definition combined immune-mediated encephalitis with toxic/metabolic encephalopathy, creating phenotypic heterogeneity whose effect on the primary finding became apparent in the sensitivity analysis.

Third, the myositis phenotype, while laboratory-anchored, used an absolute CK threshold rather than one normalized to institution-specific upper limits of normal. This approach was necessitated by the TriNetX federated architecture, which does not provide institution-specific reference ranges or enable ULN-normalized laboratory analyses [16]. However, the robustness of the myositis finding under the stringent ≥ 1000 U/L threshold in the sensitivity analysis mitigates this concern. Additionally, we could not assess the frequency of CK testing in each cohort; if ICI-treated patients underwent more frequent laboratory monitoring, this could introduce surveillance bias favoring detection of myositis in the ICI group.

Fourth, we did not account for competing risks from death, which are substantial in this advanced cancer population. High mortality rates may affect cumulative incidence estimates and could bias hazard ratios if mortality differs between groups. Fine-Gray competing risk methods would provide complementary incidence estimates.

Fifth, the study period (2014–2025) spans major practice changes in ICI uptake and supportive care. We did not include calendar time in our propensity score or outcome models, which could introduce period confounding.

Sixth, while the primary analysis retained ICI patients who subsequently received chemotherapy and excluded chemotherapy patients who later received any ICI (creating an asymmetric handling of treatment switching), the conservative sensitivity analysis applied bidirectional exclusion of treatment switching, and the primary findings for neuropathy and myositis were confirmed under this stricter specification, indicating that treatment switching did not substantially influence our conclusions.

Seventh, the primary analysis could not differentiate ICI monotherapy from combination therapy. The sensitivity analysis removed patients whose only ICI exposure was ipilimumab monotherapy by restricting inclusion to anti-PD-1 agents, though patients receiving combination nivolumab plus ipilimumab remained eligible if they also received an anti-PD-1 agent. The consistency of the myositis effect size across specifications suggests combination therapy is not the sole driver of the observed signal.

Eighth, the chemotherapy cohort encompassed agents with heterogeneous neurotoxicity profiles, including taxanes (paclitaxel), platinum compounds (carboplatin), and alkylating agents (dacarbazine, temozolomide). The TriNetX platform does not permit sequential sub-stratification within propensity score–matched cohorts while maintaining statistical power [16]. Our pooled chemotherapy neuropathy rate is therefore a blended estimate; the restricted neuropathy phenotype in the sensitivity analysis (HR 5.45) likely provides a more treatment-attributable estimate.

Ninth, we did not formally test proportional hazards assumptions across all Cox models; in the sensitivity analysis, the proportionality test for the neuropathy model was borderline, suggesting the hazard ratio may vary over time.

Tenth, the primary analysis excluded patients with brain and liver metastases to reduce confounding from concurrent radiation therapy, corticosteroids, and metabolic encephalopathy. The conservative sensitivity analysis included these patients, demonstrating that the neuropathy and myositis findings are robust to this exclusion while revealing that the encephalopathy finding does not survive inclusion of visceral metastases patients—an informative signal about the fragility of that specific outcome.

Finally, the non-convergence of the Cox model for nivolumab in the agent-level analysis reflects limited events and raises questions about statistical power for individual agent comparisons. The class effect interpretation should be considered preliminary pending adequately powered comparative studies.

### Future directions

Ongoing and future studies should prospectively characterize these neurologic adverse events using validated instruments and identify patients at greatest risk. Target trial emulation frameworks could address treatment switching and time-varying confounding more rigorously than conventional propensity score matching. Improved biomarkers—such as autoantibody profiles or T-cell repertoire characteristics—could enable earlier intervention and risk stratification. Integration of patient-reported outcome measures with EHR-based surveillance could narrow the ascertainment gap documented here. Ultimately, a better understanding of mechanisms underlying ICI-induced neurotoxicity will pave the way for targeted mitigation strategies and inform supportive care protocols tailored to modern melanoma treatment paradigms.

## Conclusions

In this propensity score–matched analysis of 13,774 melanoma patients, chemotherapy was associated with significantly higher peripheral neuropathy risk (7.2% vs 3.2%; HR, 2.47; 95% CI, 2.10–2.92), while ICI therapy was associated with higher laboratory-confirmed myositis risk (2.1% vs 1.0%; HR, 0.47; 95% CI, 0.35–0.64). A pre-specified conservative sensitivity analysis incorporating bidirectional exclusion of treatment switching, anti-PD-1 predominant ICI exposure, inclusion of brain and liver metastases, restricted outcome definitions, stringent laboratory thresholds, and 5-year follow-up confirmed and substantially strengthened the peripheral neuropathy finding (HR 5.45) and preserved the laboratory-anchored myositis finding (HR 0.46), while revealing that the modest encephalopathy signal did not survive conservative re-specification. These robust findings confirm that neurologic toxicity profiles differ fundamentally by treatment modality and support treatment-specific surveillance and supportive care strategies. Patients receiving chemotherapy should be monitored closely for peripheral neuropathy, whereas those receiving ICIs require vigilance for immune-mediated neuromuscular complications, particularly laboratory-confirmed myositis.

## Supplementary information

Below is the link to the electronic supplementary material.

ESM 1 (DOCX 561 KB)

## Data Availability

The data that support the findings of this study are available from TriNetX, LLC. Restrictions apply to the availability of these data, which were used under license for this study. Data are available from the authors upon reasonable request and with permission of TriNetX. The TriNetX platform provides access to de-identified electronic health records through a federated network; individual patient-level data cannot be exported from the platform. Researchers with institutional TriNetX access can replicate this study using the methodology described. The statistical analysis code is available upon request from the corresponding author.
