# Supplementary material for: Comparative neurologic toxicity profiles of chemotherapy versus immune checkpoint inhibitors in melanoma: a propensity score-matched analysis
Source: Support Care Cancer. 2026 May 14;34(6):538. doi: 10.1007/s00520-026-10745-4 (PMC13176077; doi:10.1007/s00520-026-10745-4)
Supplement: Supplementary file 1 — (DOCX 561 KB) [file 520_2026_10745_MOESM1_ESM.docx]

# Supplementary Material

**Manuscript:** Comparative Neurologic Toxicity Profiles of Chemotherapy Versus Immune Checkpoint Inhibitors in Melanoma: A Propensity Score-Matched Analysis of the TriNetX Global Collaborative Network

**Authors:** Majd A. AbuAlrob, Sami Alshehab, Yoused Awad, Ranim Toma, Tarneem Azzam, Rand Abdellatif, Ali Al-Salahat, Shahd Hamid

**Target Journal:** *Supportive Care in Cancer*

# Supplementary Methods: Conservative Sensitivity Analysis

## Overview

To simultaneously address reviewer concerns regarding attribution bias from treatment switching, residual confounding from combination immune checkpoint therapy, heterogeneity of the chemotherapy-induced peripheral neuropathy phenotype, variability in the myositis laboratory threshold, and limited external validity from metastatic-site exclusions, we conducted a pre-specified conservative sensitivity analysis on the TriNetX Global Collaborative Network. The analysis was run on April 10, 2026, and queried 170 healthcare organizations.

## Cohort Definitions for the Sensitivity Analysis

The sensitivity analysis used two new “no-contamination” cohorts with the following specifications:

### Cohort 1 — Chemotherapy (No Contamination)

**Inclusion criteria** (patients must meet ALL): - Age ≥18 years at the most recent occurrence - Diagnosis of melanoma on or after January 1, 2014, defined by ICD-10 codes C43.* or D03.* or ICD-O-3 morphology codes 8720/2, 8720/3, 8721/3, 8742/3, 8761/2, 8780/3, or 872–879 - At least one prescription or administration of dacarbazine, paclitaxel, carboplatin, or temozolomide (RxNorm codes 3098, 56946, 40048, 37776 respectively)

**Exclusion criteria** (patients cannot have any of the following at any time relative to the index event): - Multiple sclerosis (G35) - Small-cell lung cancer (ICD-O-3 8042/3; TriNetX SCC codeset) - Non-small cell lung cancer (TriNetX NSCC codeset) - Malignant neoplasm of bronchus and lung (C34) - Thymoma (ICD-O-3 8580/3) - Neoplasm of unspecified behavior (D49.89) - Malignant primary neoplasm, unspecified (C80.1) - Malignant neoplasm of kidney except renal pelvis (C64) - Secondary malignant neoplasm of unspecified lung (C78.00) - Pembrolizumab, nivolumab, or ipilimumab at **any time** (RxNorm codes 1547545, 1597876, 1094833; HCPCS codes J9228, J9299, C9027)

### Cohort 2 — Immune Checkpoint Inhibitor (No Contamination)

**Inclusion criteria** (patients must meet ALL): - Age ≥18 years - Diagnosis of melanoma (same codes as Cohort 1) - At least one prescription or administration of pembrolizumab or nivolumab (RxNorm codes 1547545, 1597876; HCPCS codes J9299, C9027). **Ipilimumab was removed from the inclusion criteria**, so patients whose only ICI exposure was ipilimumab monotherapy were not included.

**Exclusion criteria**: - Same non-melanoma cancer and multiple sclerosis exclusions as Cohort 1 - Dacarbazine, paclitaxel, carboplatin, or temozolomide at **any time** (before or after index ICI)

### Important note on combination therapy

The Cohort 2 inclusion requires pembrolizumab or nivolumab; ipilimumab is not in the inclusion list. However, patients who received both nivolumab and ipilimumab (combination immunotherapy) remained eligible because they met the nivolumab inclusion criterion and there was no active exclusion for ipilimumab. Therefore, the sensitivity analysis excludes patients whose **only** ICI exposure was ipilimumab monotherapy but does not fully isolate anti–PD-1 monotherapy from combination immunotherapy. We refer to the resulting cohort as “anti–PD-1 predominant” throughout the manuscript.

### Differences from the primary analysis

Key differences between the primary analysis cohorts and the sensitivity analysis cohorts are summarized below:

| Feature | Primary Analysis | Conservative Sensitivity Analysis |
| --- | --- | --- |
| Treatment switching in chemo cohort | ICI at any time → excluded | ICI at any time → excluded (same) |
| Treatment switching in ICI cohort | Chemo within 3 months **before** index → excluded; post-index chemo retained | Chemo at **any time**, before **or** after index → excluded |
| Ipilimumab in ICI inclusion | Yes | No (pembrolizumab or nivolumab only) |
| Brain metastasis exclusion | Yes | **No (included)** |
| Liver metastasis exclusion | Yes | **No (included)** |
| Observation window | 1–1,095 days (3 years) | **1–1,825 days (5 years)** |
| Peripheral neuropathy definition | Broad (G62.0, G62.2, G62.89, G63, G13.0, G56, G57.91, G57.92) | **Restricted (G62.0, G62.2 only)** |
| Myositis laboratory threshold | CK ≥500 U/L | **CK ≥1000 U/L** |
| Encephalopathy definition | G04.00, G04.81, G92, G13.1 | Same as primary |
| Fracture definition | S12–S82 codes | Same as primary |
| Propensity score matching | 1:1 nearest-neighbor, 52 covariates, automated caliper | Same |

## Propensity Score Matching in the Sensitivity Analysis

Before matching, the chemotherapy cohort comprised **10,609 patients** and the ICI cohort comprised **12,723 patients**. After 1:1 nearest-neighbor propensity score matching on the same 52 baseline covariates used in the primary analysis, **6,640 matched pairs** (13,280 patients total) were available for outcome analysis. All standardized mean differences were <0.10 after matching, and propensity score distributions showed excellent overlap between the matched cohorts.

## Follow-up Time in the Sensitivity Analysis

After matching, the mean follow-up was 640.1 ± 593.6 days (median 435, IQR 775) in the chemotherapy cohort and 739.9 ± 623.3 days (median 552, IQR 1,033) in the ICI cohort, reflecting the 1,825-day (5-year) observation window.

## Statistical Analysis

Statistical methods were identical to the primary analysis: three complementary analytic approaches (measures of association, Kaplan–Meier time-to-event analysis with log-rank testing, and number-of-instances analysis for recurrent events) were applied to each outcome. Hazard ratios, risk ratios, and odds ratios are reported as chemotherapy vs ICI. Patients with the outcome prior to the observation window were excluded from the specific outcome analysis to isolate new-onset cases.

# Supplementary Table S1. Primary Analysis vs Conservative Sensitivity Analysis — Complete Results

All hazard ratios and risk ratios are reported as Chemotherapy vs ICI. Values >1 indicate higher risk with chemotherapy; values <1 indicate higher risk with ICI.

## Matched Cohort Sizes

| Analysis | Chemo Before PSM | ICI Before PSM | Matched Pairs |
| --- | --- | --- | --- |
| Primary Analysis (3-year follow-up) | 9,787 | 14,627 | 6,887 |
| Conservative Sensitivity Analysis (5-year follow-up) | 10,609 | 12,723 | 6,640 |

## Peripheral Neuropathy

| Metric | Primary Analysis | Conservative Sensitivity Analysis |
| --- | --- | --- |
| Outcome definition | Broad: G62.0, G62.2, G62.89, G63, G13.0, G56, G57.91, G57.92 | Restricted: G62.0, G62.2 only |
| Observation window | 1–1,095 days | 1–1,825 days |
| Chemo — events / at-risk | 446 / 6,188 | 304 / 6,486 |
| ICI — events / at-risk | 205 / 6,408 | 63 / 6,559 |
| Chemo — cumulative incidence | 7.2% (3 years) | 4.7% (5 years) |
| ICI — cumulative incidence | 3.2% (3 years) | 1.0% (5 years) |
| Risk Difference (95% CI) | — | 0.037 (0.032–0.043) |
| Risk Ratio (95% CI) | 2.25 (1.92–2.65) | **4.88 (3.73–6.39)** |
| Odds Ratio (95% CI) | — | 5.07 (3.86–6.67) |
| Chemo outcome-free survival at end of window | 88.7% | 92.2% |
| ICI outcome-free survival at end of window | 94.7% | 98.2% |
| Log-Rank χ² | 123.1 | 189.1 |
| **Hazard Ratio (95% CI)** | **2.47 (2.10–2.92)** | **5.45 (4.16–7.15)** |
| P value | <.001 | <.001 |
| Patients excluded for prior outcome (Chemo / ICI) | — | 154 / 81 |

## Encephalopathy

| Metric | Primary Analysis | Conservative Sensitivity Analysis |
| --- | --- | --- |
| Outcome definition | G04.00, G04.81, G92, G13.1 | Same as primary |
| Observation window | 1–1,095 days | 1–1,825 days |
| Chemo — events / at-risk | 67 / 6,823 | 104 / 6,577 |
| ICI — events / at-risk | 99 / 6,833 | 111 / 6,596 |
| Chemo — cumulative incidence | 1.0% (3 years) | 1.6% (5 years) |
| ICI — cumulative incidence | 1.4% (3 years) | 1.7% (5 years) |
| Risk Difference (95% CI) | — | −0.001 (−0.005 to 0.003) |
| Risk Ratio (95% CI) | 0.68 (0.50–0.92) | **0.94 (0.72–1.23)** |
| Odds Ratio (95% CI) | — | 0.94 (0.72–1.23) |
| Chemo outcome-free survival at end of window | 98.7% | 96.8% |
| ICI outcome-free survival at end of window | 97.7% | 97.2% |
| Log-Rank χ² | 5.09 | 0.04 |
| **Hazard Ratio (95% CI)** | **0.70 (0.51–0.96)** | **1.03 (0.79–1.34)** |
| P value | .014 | .844 |
| Patients excluded for prior outcome (Chemo / ICI) | — | 63 / 44 |

## Myositis (Laboratory-Anchored)

| Metric | Primary Analysis | Conservative Sensitivity Analysis |
| --- | --- | --- |
| Outcome definition | ICD-10 myositis codes + CK ≥500 U/L | ICD-10 myositis codes + CK **≥1000 U/L** |
| Observation window | 1–1,095 days | 1–1,825 days |
| Chemo — events / at-risk | 64 / 6,666 | 51 / 6,489 |
| ICI — events / at-risk | 139 / 6,624 | 119 / 6,457 |
| Chemo — cumulative incidence | 1.0% (3 years) | 0.8% (5 years) |
| ICI — cumulative incidence | 2.1% (3 years) | 1.8% (5 years) |
| Risk Difference (95% CI) | — | −0.011 (−0.014 to −0.007) |
| Risk Ratio (95% CI) | 0.46 (0.34–0.61) | **0.43 (0.31–0.59)** |
| Odds Ratio (95% CI) | — | 0.42 (0.30–0.59) |
| Chemo outcome-free survival at end of window | 98.3% | 98.5% |
| ICI outcome-free survival at end of window | 96.8% | 97.0% |
| Log-Rank χ² | 25.7 | 22.9 |
| **Hazard Ratio (95% CI)** | **0.47 (0.35–0.64)** | **0.46 (0.33–0.64)** |
| P value | <.001 | <.001 |
| Patients excluded for prior outcome (Chemo / ICI) | — | 151 / 183 |
| Mean recurrent myositis episodes per affected patient (Chemo / ICI) | — | 1.53 / 3.83 |

## Fractures (Negative Control)

| Metric | Primary Analysis | Conservative Sensitivity Analysis |
| --- | --- | --- |
| Outcome definition | S12, S22, S32, S42, S52, S62, S72, S82 | Same as primary |
| Observation window | 1–1,095 days | 1–1,825 days |
| Chemo — events / at-risk | 255 / 6,099 | 253 / 5,863 |
| ICI — events / at-risk | 292 / 6,271 | 268 / 6,085 |
| Chemo — cumulative incidence | 4.2% (3 years) | 4.3% (5 years) |
| ICI — cumulative incidence | 4.7% (3 years) | 4.4% (5 years) |
| Risk Difference (95% CI) | — | −0.001 (−0.008 to 0.006) |
| Risk Ratio (95% CI) | 0.90 (0.76–1.06) | **0.98 (0.83–1.16)** |
| Odds Ratio (95% CI) | — | 0.98 (0.82–1.17) |
| P value | .20 | .812 |
| Patients excluded for prior outcome (Chemo / ICI) | — | 777 / 555 |

1. encephalopathy (G92) and paraneoplastic CNS involvement (G13.1). This heterogeneous phenotype is particularly susceptible to confounding from non-treatment-related etiologies.
